# Lack of efficacy of triclabendazole against Fasciola hepatica is present on sheep farms in three regions of England, and Wales

**DOI:** 10.1136/vr.105209

**Published:** 2019-03-01

**Authors:** Juriah Kamaludeen, John Graham-Brown, Nathalie Stephens, Josephine Miller, Alison Howell, Nicola J Beesley, Jane Hodgkinson, Jane Learmount, Diana Williams

**Affiliations:** 1 Faculty of Agriculture and Food Sciences, Universiti Putra Malaysia—Kampus Bintulu, Bintulu, Malaysia; 2 Infection Biology, University of Liverpool Institute of Infection and Global Health, Liverpool, UK; 3 Animal and Plant Health Agency, Sand Hutton, UK; 4 University of Liverpool School of Veterinary Science, Liverpool, UK; 5 Infection Biology, University of Liverpool, Liverpool, UK; 6 United Kingdom Department for Environment Food and Rural Affairs, London, UK; 7 University of Liverpool Institute of Infection and Global Health, Liverpool, UK

**Keywords:** fasciola hepatica, triclabendazole, resistance, cfecrt, spatial analysis, sheep

## Abstract

The liver fluke *Fasciola hepatica* is a parasitic trematode that has a major impact on livestock production and human health. Control of *F hepatica* is difficult and relies on anthelmintics, particularly triclabendazole, due to its efficacy against both adult and juvenile stages of the parasite. Emergence of triclabendazole-resistant *F hepatica* populations has been reported in a number of countries, including the UK, but the overall prevalence and distribution of triclabendazole resistance is unknown. In this study, the authors established the presence of reduced efficacy of triclabendazole in sheep flocks in England and Wales, using a validated composite faecal egg count reduction test. Seventy-four sheep farms were sampled from Wales, southwest, northwest and northeast England between Autumn 2013 and Spring 2015. *F hepatica* eggs were detected in samples from 42/74 farms. Evidence of a lack of efficacy of triclabendazole was detected on 21/26 farms on which the faecal egg count reduction test was completed, with faecal egg count reductions ranging from 89 per cent to 0per cent. Regression analysis suggested that both prevalence of *F hepatica* and lack of efficacy of triclabendazole were spatially correlated, with higher faecal egg counts and lower percentage reductions on farms located in the northwest of England, and Wales. Overall, the results show that reduced efficacy of triclabendazole is present across England and Wales, with a complete lack of therapeutic efficacy observed on 9/26 farms.

## Introduction

*Fasciola hepatica* is a parasitic trematode of one health importance due to its global distribution and ability to infect multiple host species, including humans. It has a negative impact on food security through its effect on livestock productivity. In sheep and cattle, effects of infection range from clinical disease, with high levels of mortality and morbidity, to long-standing subclinical infections which reduce animal productivity, growth and fertility.^1–3^ Furthermore, human fasciolosis is considered a re-emerging neglected tropical disease, with estimates of 2.6–17 million individuals infected with *Fasciola* species and a further 91 million people at risk globally.^4–6^

In temperate regions, including the UK, prevalence of liver fluke in livestock has been increasing both spatially and temporally in recent years.^7 8^ Such increases are largely attributed to changes in climatic conditions which favour the development of free-living stages of the parasite and its molluscan intermediate host, in Europe *Galba truncatula*.^9 10^

Control of fasciolosis is heavily reliant on the use of anthelmintic drugs, of which there are a limited number. Of these, the pro-benzimidazole triclabendazole (TCBZ) is commonly used, due to its unique efficacy against all stages of the parasite^11^; in sheep, TCBZ is effective at killing parasites from 2 days postinfection onwards.^12^ TCBZ-resistant (TCBZ-R) *F hepatica* populations have been reported on multiple occasions across Europe, Australasia and South America.^13–18^ TCBZ-R has also been documented in a case of human infection, with evidence to suggest this arose from TCBZ-R parasites infecting livestock in the locality.^19^

Neither the mode of action of TCBZ against *F hepatica* nor the mechanisms of resistance are known, and no new drugs or vaccines are likely to reach the market in the near future. A recent study examining the population structure of *F hepatica* in the UK demonstrated high levels of genetic diversity, a lack of population structure and high gene flow, suggesting resistance genes have the potential to spread rapidly within populations of *F hepatica*.^20^ It is therefore important that the extent of TCBZ resistance is known at a regional level, so that advice on quarantine treatments, and strategic use of TCBZ as part of an overall control programme for *F hepatica*, can be developed for the industry.

Here, the authors describe the results of a study to establish the presence and distribution of TCBZ resistance in four major sheep rearing regions of Great Britain, namely northwest (NW) England, northeast (NE) England, southwest (SW) England and Wales, using the composite faecal egg count reduction test (cFECRT) described by Daniel and others.^21^

## Methods

### Farm recruitment and sampling protocol

Farms were recruited into the study from three sources:

1. In NW England, farms were recruited through the Cumbria Farmers Network (CFN), an independent not-for-profit farming support network formed in 2006 which, at the time this study was conducted, had over 500 members (www.thefarmernetwork.co.uk).
2. Names and addresses of 750 sheep farms from NE England, SW England and Wales were supplied by the Rapid Analysis and Detection of Animal-related Risks (RADAR) database. A random selection of 250 farms were invited to participate.
3. An additional 12 farms that were part of an Animal and Plant Health Agency (APHA) study investigating sustainable nematode control.^22^ These Sustainable Control of Parasites in Sheep (SCOPS) study farms were located in Wales, SW and NE England.

The fluke infection status of study farms was unknown before their recruitment. Farmers were either contacted by post (RADAR database) or phone (CFN and SCOPS farms). All participants were then sent an information sheet and asked to sign a consent form ahead of enrolment.

Farms recruited through CFN were sampled between August and November 2013. Those farms recruited through the RADAR database and SCOPS study were sampled between December 2014 and April 2015.

Farm visits by veterinary or APHA staff were arranged a minimum of 12 weeks after any previous flukicidal treatment. A validated cFECRT was used to estimate TCBZ treatment efficacy^21^; briefly, 20 randomly selected sheep from the same management group were gathered and penned in two groups of 10. After about 20 minutes, the sheep were released and 10 individual faecal samples were collected from the floor of each pen. The weight of each sheep was measured and they were treated with TCBZ (FASINEX 5 per cent, Novartis, 10 mg/kg/animal orally). If farms had a positive pretreatment egg count, the same 20 individuals were resampled at 21 days post-treatment. However, only data from farms with a pretreatment egg counts of greater than equal to 100 eggs per 100 g, that is, ≥1 epg, of faeces were used to calculate percentage faecal egg count reduction (%FECR), this being the previously established threshold.^21^ Results were sent back to each farm as soon as they were available.

All farm data were anonymised and stored in accordance with the UK Data Protection Act (1998).

### Composite faecal egg sedimentation

Composite faecal samples were prepared from 10×5 g individual samples to give a total of 50 g. Faeces were homogenised in water and washed through stacked test sieves with decreasing apertures (710, 150 and 38 µm, respectively). The material retained in the 38 µm sieve was transferred to a 500 ml glass beaker, diluted to 500 ml in water and left to stand for 4 minutes to allow sedimentation of fluke eggs. After this time water was poured off leaving a slurry of about 50 ml. The process was repeated until the supernatant was clear after the 4 minutes sedimentation period. The sediment was transferred to a large petri dish, optionally stained with two to three drops of 10 per cent methylene blue, examined under a low power dissecting microscope (10x to 40x magnification) and the total number of *F hepatica* eggs recorded. In some samples, paramphistome (rumen fluke) eggs were observed. Distinction between egg types was made visually: *F hepatica* eggs have a golden brown coloration while parampistome eggs have a clear, uncolored appearance. Paramphistome eggs were not included in the final fluke egg counts.

The counts for the two composites were combined to give a total egg count per 100 g. Percentage faecal egg count reduction was only calculated if the pretreatment egg count was greater than equal to 1 epg using the following formula:

$$\%FECR\;=\;100-\;\left(\left(\frac{post\text{-}treatment\;count}{pre\;treatment\;count}\right)\;\times 100\right)$$

A lack of drug efficacy was assumed if %FECR was less than 90 per cent.

### Statistical analysis

To identify associations both for presence of *F hepatica* infection and efficacy of TCBZ treatment, results were compared with spatial and temporal explanatory variables by linear regression analysis.

Since composite fluke egg counts have been shown to correlate positively with group prevalence,^21 23^ counts from the pretreatment sample collected from all farms (n=74) were used as an indicator of prevalence within each flock. Fluke egg counts were modelled as a response variable (*Y*) and compared with the following explanatory variables (*x*): (i) farm GPS coordinates to assess the spatial distribution and (ii) a binomial term distinguishing sampling period for each farm (2013 or 2014/15) to allow potential variation in pasture infectivity between fluke seasons to be considered (autumn 2013 vs winter 2014/15). These explanatory variables were included simultaneously in a multivariable regression analysis. Before this, Box-Cox analysis determined a log(+1) transformation of the response variable (*Y*) was the most appropriate measure to ensure linear fit.^24^

Twenty-six fluke egg positive farms had initial fluke egg counts greater than equal to 100 and were resampled at 21 days post-TCBZ treatment. To investigate associations with reduced TCBZ efficacy using linear regression analysis, percentage reduction in egg count was calculated for those farms. For this analysis, where post-treatment egg counts exceeded pretreatment values, percentage reduction was taken to be zero. To avoid overparameterising this smaller dataset univariable analysis was performed, with log(+1) transformation of per cent reduction used as the response variable (*Y*) against each of the explanatory variables (*x*) described above.

All models were checked for goodness of fit by normality and residual plots. Results were interpreted from the mean and standard error (SE) of the coefficient (β) and P value estimated for each explanatory variable (*x*).

## Results

A total of 74 farms were recruited to the study (table 1): 20 farms were approached through the CFN, with 16 farms ultimately sampled between August and November 2013. Forty-six farms from RADAR and 12 from the SCOPS study were recruited and sampled between December 2014 and April 2015.

**Table 1:** Number and regional distribution of sampled and fluke egg positive farms at initial recruitment and at 21 days post-treatment with TCBZ

	Pretreatment sampling			Post-treatment sampling: number of farms with <90% egg count reduction*
	Number of farms sampled	Fluke positive (1–100 eggs per 100 g)	Fluke positive (≥100 eggs per 100 g)*	
NW England	17	3	13	13
Wales	17	4	3	2
SW England	17	5	0	NA
NE England	23	4	10	6
**Total**	**74**	**16**	**26**	**21**

Counts were based on two composite faecal samples, each comprised 10x 5 g individual samples. For repeated composite counts post-TCBZ treatment, faecal samples were taken from the same individuals sampled at pre treatment.

*Only farms where pretreatment counts were ≥100 eggs per 100 g (≥1 epg) were used to calculate faecal egg count reduction post-treatment.

NE, northeast; NW, northwest; SW, southwest; TCBZ, triclabendazole.

**Figure 1:**
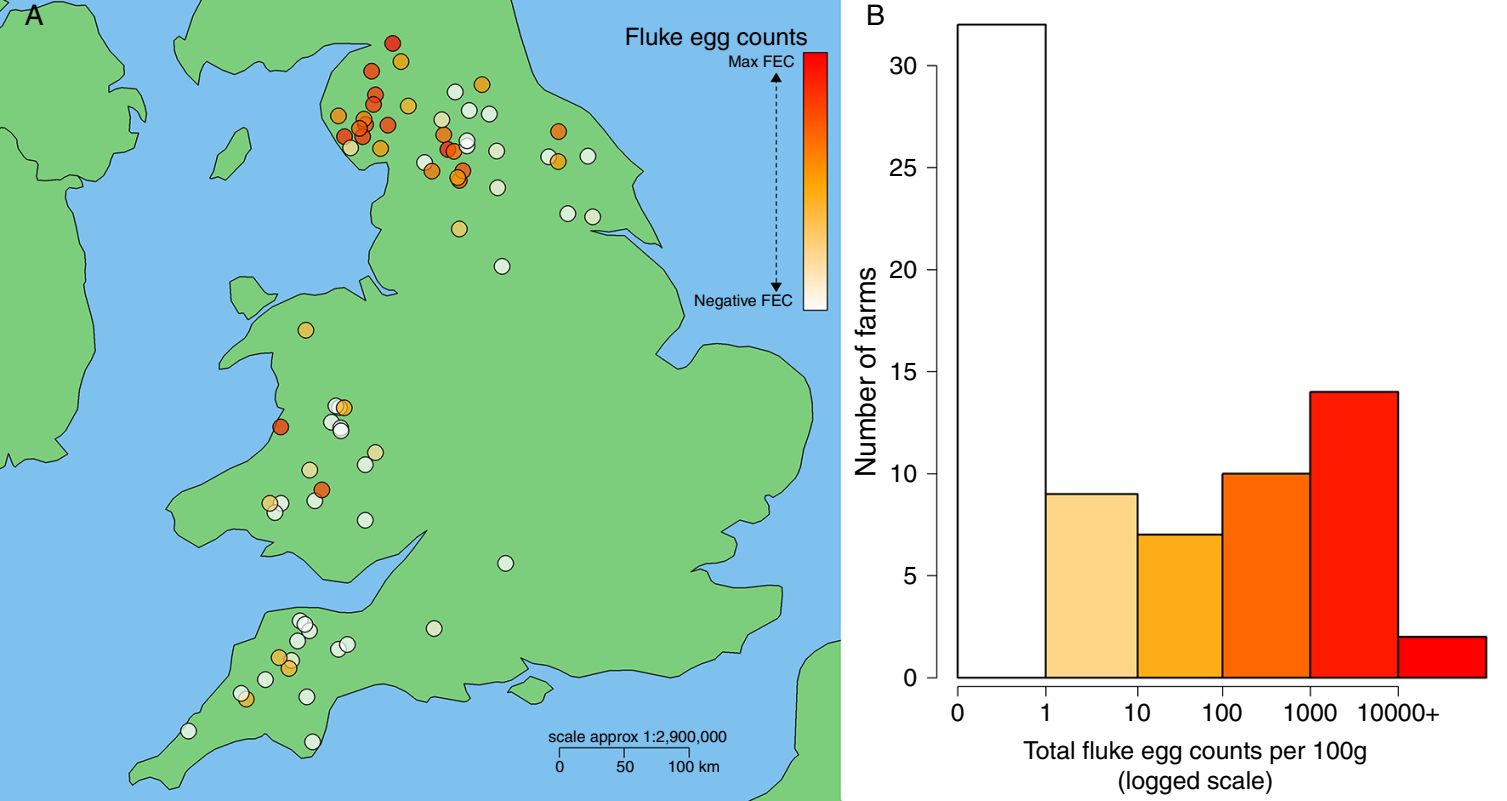
Composite faecal egg counts per 100 g faeces showing (A) spatial distribution of individual farms sampled (n=74) and (B) range of recorded composite counts. Colour in both plots correspond to recorded egg counts per 100 g, with white signifying an egg count of zero and orange to red demonstrating the observed range in positive counts from 1 to 21,664 eggs per 100 g faeces. Spatial coordinates jittered 0.15^o^ by 0.09^o^ latitude/longitude (approximately 10 km^2^).

Positive faecal egg counts were obtained for 42 of the 74 farms sampled; counts ranged from 1 to greater than 20,000 eggs per 100 g faeces (table 1 and figure 1).

**Figure 2:**
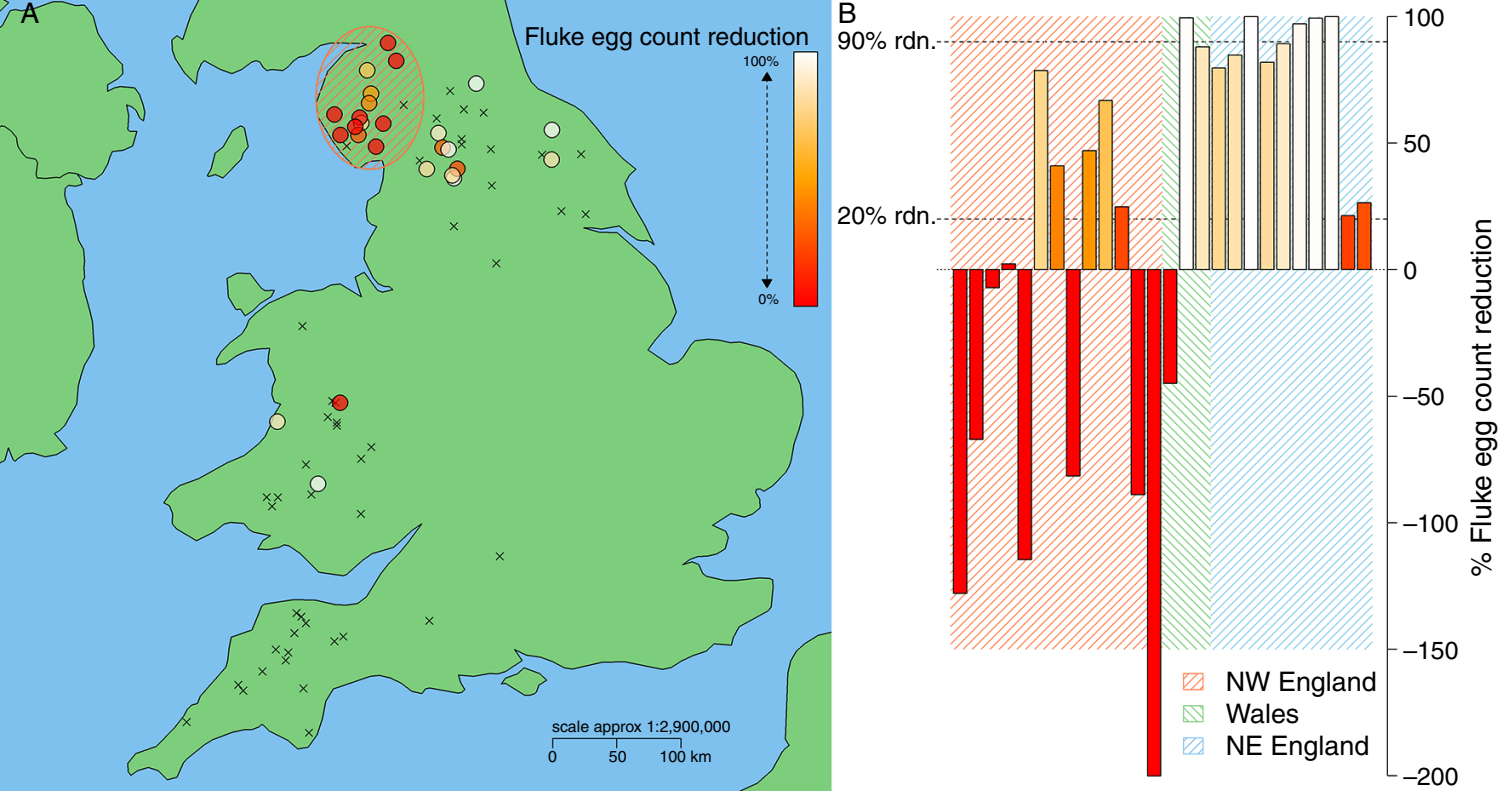
Percentage faecal egg count reduction (%FECR) at resampling 21 days post-treatment with TCBZ with (A) spatial distribution and (B) per cent reductions for individual farms and regions. %FECR were calculated for post-treatment positive farms with initial egg counts ≥100 fluke eggs per 100 g faeces (n=26). Colour in both plots corresponds to %FECR, with white to red signifying range in %FECR from 100% to 0%. For regression analysis, all negative percentage reductions (farms where counts increased at 21 day resampling) were taken to be zero. x indicates an absence of %FECR data on farms where initial composite counts of <100 eggs per 100 g of faeces were recorded. Hatched regions in (B) denote the categorisation of individual farms by region, and highlight farms from northwest (NW) England in (A). Spatial coordinates jittered 0.15^o^ by 0.09^o^ latitude/longitude (approximately 10 km^2^).

Twenty-six farms had pretreatment egg counts of greater than equal to 100, and the %FECR was calculated. Of these, 21 farms showed evidence of a reduction in drug efficacy. Twelve farms showed a reduction ranging from 89 per cent to 20 per cent and, on nine farms, TCBZ was completely ineffective, with post-treatment egg counts remaining the same or increasing (table 1 and figure 2).

Both the proportion of infected flocks and pretreatment faecal egg counts were highest in NW England, followed by Wales (figure 1). Multivariable linear regression analysis of (log(+1) transformed) pretreatment fluke egg counts revealed a strong spatial correlation, with a positive relationship found between egg counts and coordinates of increasing (northerly) latitude and decreasing (westerly) longitude (table 2). Year in which samples were collected was not found to be statistically significant in this multivariable analysis (table 2).

**Table 2:** Multivariable linear regression results for the response variable (*Y*) faecal egg count (log(+1) transformed)

Model ID	Explanatory variable (*x*)	Coefficient value (β)	SE	P value
Multi_lm_1	Spatial coordinates:			
	Longitude (easting)	−1.232	0.541	0.026*
	Latitude (northing)	1.486	0.423	<0.001†
	2013 vs 2014/15	−1.763	1.129	0.123

Coefficients (β) indicate the relationship between each explanatory variable (x) and the response variable (*Y*) with associated standard error (SE) and P value.

*Signifies P<0.05 (95% CI).

†Signifies P<0.005 (99.5% CI).

The FECRT was used on farms in three regions. Most of the farms where a lack of TCBZ efficacy was observed were situated in NW England (figure 2). Univariable spatial analysis of (log(+1) transformed) per cent FECR, with a statistically significant (P<0.05) relationship between decreasing percentage egg count reduction and decreasing (westerly) longitude (table 3). Samples collected in 2014/15 were also observed to have a higher percentage reduction when compared with those collected in 2013 (table 3).

**Table 3:** Univariable linear regression results for the response variable (*Y*) percentage faecal egg count reduction (log(+1) transformed)

Model ID	Explanatory variable (*x*)	Coefficient value (β)	SE	P value
Uni_lm_1	Spatial coordinates:			
	Longitude (easting)	1.519	0.522	0.008*
	Latitude (northing)	−1.028	0.523	0.061
Uni_lm_2	2013 vs 2014/15	−2.394	0.648	0.001†

Coefficients (β) indicate the relationship between each explanatory variable (*x*) and the response variable (*Y*) with associated standard error (SE) and P value.

*Signifies P<0.01 (99% CI).

†Signifies P<0.005 (99.5% CI).

None of the farms tested in SW England had pretreatment egg counts that met the minimum criteria of greater than equal to 1 epg faeces. However, the two farms in that region with the highest positive egg counts (78 eggs/100 g and 45 eggs/100 g, respectively) when resampled 21 days post-treatment recorded faecal egg counts of 51 and 12 eggs/100 g faeces, respectively, providing some evidence that TCBZ was not fully effective.

## Discussion

This study is the first of its kind to demonstrate the presence and distribution of reduced TCBZ efficacy across two of the world’s leading sheep rearing countries (England and Wales). Of the 74 farms that participated in the study, 42 had positive fluke egg counts. A reduction in the efficacy of TCBZ was detected on 21 of 26 farms on which the full cFECRT was completed. On nine of those farms, egg counts either remained at pretreatment levels or increased 21 days after treatment, suggesting complete drug failure. Encouragingly, while reduced efficacy was demonstrated, TCBZ had some effect on egg counts on 12 farms. Linear regression analysis showed that, where the cFECRT was performed, reduced efficacy was most likely to occur in the more westerly parts of the UK, and that significantly higher fluke egg counts were also found in these regions, particularly NW England.

Based on the cFECRT, evidence of reduced TCBZ efficacy was detected on farms in NW England, NE England and Wales. None of the farms tested in SW England had pretreatment egg counts that met the minimum criteria of 1 epg faeces.^21^ However, while they did not contribute per cent FECR data, the two farms in that region with the highest positive egg counts did provide evidence suggesting that TCBZ was not fully effective. This led us to the conclusion that reduced efficacy of TCBZ was evident in Wales and all three regions of England investigated.

Thirteen of the 21 farms where reduced efficacy was detected were from NW England. Our multivariable regression analysis suggested that farms in the north and west were more likely to have higher FECs, while NW England region has previously been shown to have the highest fluke prevalence of anywhere in England and Wales.^25 26^ Hence, it is likely that in such areas use of flukicides like TCBZ would be more extensive and the selection pressure for resistance will be high. It is important to note, however, evidence of reduced TCBZ efficacy was also detected in regions typically considered to be of low or medium risk of fluke infection also (NE and SW England, respectively). It is suggested that animal movements, which have increased in recent years, have helped introduce *F hepatica* into areas of the UK where it is not traditionally found, particularly if farms are involved in agrienvironment schemes promoting wetland management.^27^ Movement of animals may also introduce TCBZ-resistant parasites into areas where drug selection pressure may be low and therefore resistance not suspected.^28^ We have recently shown that there are high levels of genetic diversity, a lack of population structure and high gene flow in populations of *F hepatica*, suggesting that resistance genes have the potential to spread rapidly within and between populations of parasites affecting both sheep and cattle.^20^ These results therefore highlight the importance of testing and quarantine dosing sheep and cattle when they are moved onto a farm.

Participation in the study demanded considerable time and effort by farmers. In order to maximise recruitment, the authors therefore identified and approached suitable candidates through several routes, namely a farmers’ cooperative in NW England, the RADAR database and the SCOPS study being conducted by the APHA. While pre-existing knowledge of a farm’s fluke infection status was not a prerequisite for enrolment, it is possible that a number of the farmers who responded to the invitations may have already had evidence of fluke infection on their farms and/or suspected resistance to TCBZ. Consequently, potential for bias in this cohort cannot be excluded, meaning the proportion of farms demonstrating a lack of TCBZ efficacy should not necessarily be considered as representative of either the UK as whole, or its constituent regions. Similarly, the different methods of recruitment and the fact that the farms were tested over three different years could have introduced bias, particularly as the majority of farms from NW England were sampled in 2013 following the unusually wet year of 2012.^29 30^

For this study the authors used a validated cFECRT.^21^ This is a useful field test that has been developed to evaluate TCBZ use in sheep. It does not prove that resistance is present, but demonstrates whether TCBZ is effective in reducing the faecal egg counts. Aside from indicating TCBZ resistance, alternative explanations for reduction in efficacy may relate to incorrect (under-)dosing, faulty drenching equipment, etc. However, the trials conducted here were carried out by qualified staff, dosing guns were correctly calibrated, sheep were weighed and samples were collected according to the prescribed protocol. In the absence of other recommended protocols,^31^ the authors consider this test to be a useful tool in assessing TCBZ efficacy on farms.

In conclusion, this study demonstrates the spatial distribution of reduced triclabendazole efficacy in two major sheep producing countries of the world. Of 74 farms tested, 21 showed evidence of reduced drug efficacy, suggesting TCBZ resistance is widespread and demonstrating the potential for this to become a major problem for sheep producers worldwide. These results also highlight the importance of promoting sustainable parasite control techniques in order to reduce selection for drug resistance and preserve TCBZ where it is still effective.
